# A Double-Blind, Placebo-Controlled Trial to Assess Safety and Tolerability of (Thetanix) *Bacteroides thetaiotaomicron* in Adolescent Crohn's Disease

**DOI:** 10.14309/ctg.0000000000000287

**Published:** 2020-12-18

**Authors:** Richard Hansen, Ian R. Sanderson, Rafeeq Muhammed, Stephen Allen, Christos Tzivinikos, Paul Henderson, Lisa Gervais, Ian B. Jeffery, David P. Mullins, Eileen A. O'Herlihy, John D. Weinberg, Geoff Kitson, Richard K. Russell, David C. Wilson

**Affiliations:** 1 Paediatric Gastroenterology, Royal Hospital for Children, Glasgow, UK;; 2 Blizard Institute, Queen Mary University of London, London, UK;; 3 Paediatric Gastroenterology, Birmingham Children's Hospital, Birmingham, UK;; 4 Department of Clinical Sciences, Liverpool School of Tropical Medicine, Liverpool, UK;; 5 Paediatric Gastroenterology, Alder Hey Children's Hospital, Liverpool, UK;; 6 Paediatric Gastroenterology and Nutrition, Royal Hospital for Sick Children, Edinburgh, UK;; 7 Child Life and Health, University of Edinburgh, Edinburgh, UK;; 8 4D pharma Cork Limited, University College Cork, Cork, Ireland;; 9 4D pharma, Leeds, UK;; 10 ProPharma Partners Limited, Horsham, UK.

## Abstract

**INTRODUCTION:**

Thetanix (gastroresistant capsules containing lyophilized *Bacteroides thetaiotaomicron*) is a live biotherapeutic, under development for Crohn's disease, that antagonizes transcription factor nuclear factor kappa B, reducing proinflammatory cytokines, particularly tumor necrosis factor alpha. We aimed to assess safety and tolerability in adolescents with Crohn's disease in remission.

**METHODS:**

Subjects who were 16–18 years with Crohn's in remission (weighted pediatric Crohn's disease activity index <12.5) were recruited. Each active dose comprised ∼10^8.2±1.4^ colony forming units of *B. thetaiotaomicron* (randomized 4:1 active:placebo). Part A was single dose. Part B involved 7.5 days twice daily dosing. Serial stools were analyzed for calprotectin, 16S rRNA sequencing, and *B. thetaiotaomicron* real-time polymerase chain reaction. Bloods were taken serially. Subjects reported adverse events and recorded temperature twice daily.

**RESULTS:**

Fifteen subjects were treated—8 in part A (75% men, median 17.1 years) and 10 in part B, including 3 from part A (80% men, median 17.1 years); all 18 completed. Seventy percent took concurrent immunosuppression. Reported compliance was >99% in part B. Two subjects reported adverse events deemed related—one in part A with eructation, flatulence, and reflux; one in part B with dizziness, abdominal pain, and headache. No serious adverse events were reported. There was no significant change in median calprotectin across part B (87.8 [4.4–447] to 50.5 [5.3–572], P = 0.44 by the Fisher exact test in the active group). No significant differences were found in microbiota profiles, but diversity seemed to increase in treated subjects.

**DISCUSSION:**

Thetanix, after single and multiple doses, was well tolerated. Although the numbers in this study were small, the safety profile seems good. Future studies should explore efficacy.

## INTRODUCTION

We stand on the cusp of a potential therapeutic revolution in inflammatory bowel disease (IBD) management centered on microbial therapeutics (1,2). The genetic revolution in IBD pointed firmly to the microbiome as pivotal in disease pathogenesis, with the seminal study by Jostins and colleagues stating “Most of the evidence relating to possible causal genes points to an essential role for host defense against infection in IBD” (3). Despite this, and progress with fecal microbial transplantation as a treatment for ulcerative colitis (4,5) and selected antibiotics (6) and nutritional therapy in Crohn's disease (CD) (7–9), data on probiotics as therapy for CD have been disappointing to date (10,11). One published editorial suggested that the reason for this disappointment might be because we have not tested the correct organisms, the correct combination of organisms, the correct subsets of patients, or perhaps even because our understanding of the host–microbe interactions in IBD is flawed (12). Indeed, most probiotics tested to date tend to have been selected for convenience or ease of manufacture, rather than biological plausibility. There is, therefore, a need to revisit the potential for targeted microbial therapy using organisms that may have more relevance to the disease, accepting that they may be commercially harder to work with, including *Bacteroides*, *Faecalibacterium,* and *Akkermansia* species (12). This approach has been termed live biotherapeutics or next-generation probiotics.

*Bacteroides thetaiotaomicron* is one such species—a nonmotile, purely anaerobic symbiotic bacterium—with the ability to digest dietary fibers and host glycans while producing short chain and organic acids, and a cell surface that both interacts with and evades the host immune system (13). The organism had a sequencing read prevalence of ∼1.5% in a recent longitudinal pediatric CD microbiome study (14) and has been shown to support and stimulate mucus production within the colon, which may enhance an important innate immune mechanism against bacterial invasion (15). *B. thetaiotaomicron* also attenuates gut inflammation via enhancement of nuclear factor kappa B (NF-κB) subunit RelA nuclear export and subsequent antagonism of transcription factor NF-κB (16). The pivotal importance of NF-κB in driving tumor necrosis factor alpha (TNFα) release after NOD2 expression highlights the importance of this transcription factor in CD immunopathology (17). *B. thetaiotaomicron* therefore has potential as a live biotherapeutic and mucosally active anti-TNFα.

In 2 rodent models of colitis (dextran sodium sulphate [DSS] and interleukin-10 knockout), *B. thetaiotaomicron* ameliorated weight loss, histological damage, and immunological activation, including reduced expression of TNFα in the DSS model (18). Importantly, lyophilized *B. thetaiotaomicron* maintained similar levels of efficacy to actively growing bacteria in ameliorating DSS colitis, suggesting that a lyophilized preparation could prove useful in treating gastrointestinal inflammation in humans.

Our study's aim was to assess the safety and tolerability of *B. thetaiotaomicron* in patients with CD; therefore, we chose to perform a first-in-humans safety study of lyophilized *B. thetaiotaomicron* (Thetanix) in adolescent patients with known CD in clinical remission. We opted to test the investigational medicinal product (IMP) in patients with CD first because the abundance of *B. thetaiotaomicron* in the healthy gut microbiome would likely make a healthy volunteer study redundant. This position was agreed with the United Kingdom's Medicines and Healthcare Products Regulatory Agency before our submission to the regional ethics committee.

## MATERIALS AND METHODS

This was a randomized, double-blind, placebo-controlled study in subjects with CD in remission, aged 16–18 years. The study started with a single-dose challenge with follow-up for adverse events (AEs) (part A, n = 8) and then proceeded to part B, in which twice daily dosing was provided for 7.5 days (n = 10). Patients who participated in part A were also allowed to participate in part B. Subjects suitable for this study were identified from current patient lists at pediatric gastroenterology clinics in Royal Hospital for Children, Glasgow; Royal Hospital for Sick Children, Edinburgh; Birmingham Children's Hospital; Alder Hey Children's Hospital, Liverpool; and Royal London Hospital. Inclusion and exclusion criteria were devised to identify patients with stable CD in remission during late adolescence and were subsequently amended to allow patients stable on anti-TNFα therapy and after resection surgery to be included (Box 1). A urine pregnancy test was completed for all female subjects who were postmenarche. The recruitment target for both part A and part B was set at 10 participants, with inclusion in both parts acceptable if inclusion/exclusion criteria could still be achieved after rescreening. The recruitment target to part A was revised to 8 subjects after a substantial amendment. A review of all safety data was undertaken by the safety review committee (SRC), comprising the coordinating investigator, the principle investigators for active sites, site coinvestigators, and the medical monitor, before progression to part B was deemed appropriate.

**Box 1.**
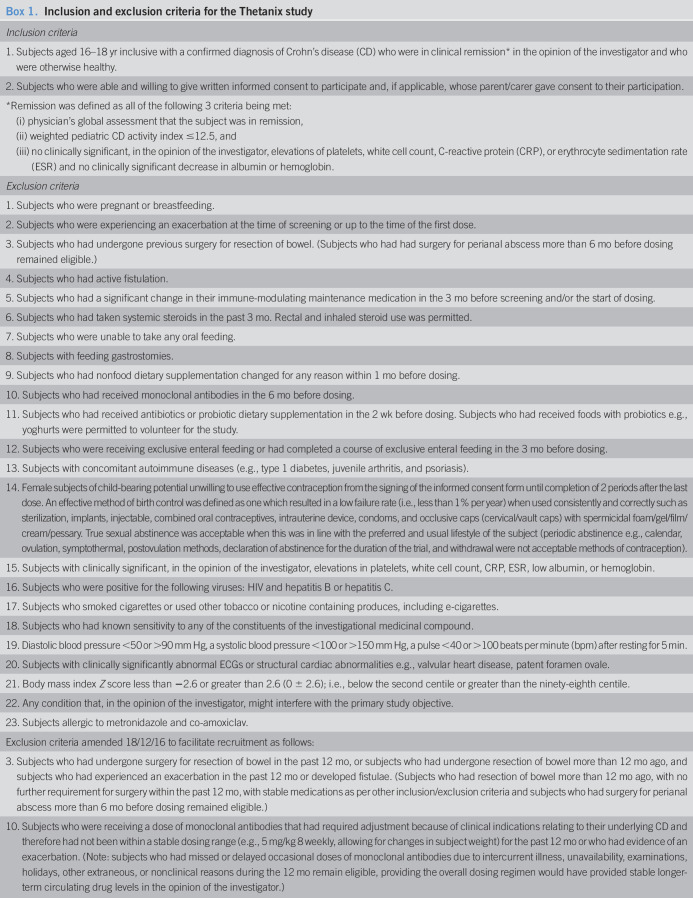
Inclusion and exclusion criteria for the Thetanix study

*Inclusion criteria*
1. Subjects aged 16–18 yr inclusive with a confirmed diagnosis of Crohn's disease (CD) who were in clinical remission* in the opinion of the investigator and who were otherwise healthy.
2. Subjects who were able and willing to give written informed consent to participate and, if applicable, whose parent/carer gave consent to their participation.
*Remission was defined as all of the following 3 criteria being met: (i) physician's global assessment that the subject was in remission, (ii) weighted pediatric CD activity index ≤12.5, and (iii) no clinically significant, in the opinion of the investigator, elevations of platelets, white cell count, C-reactive protein (CRP), or erythrocyte sedimentation rate (ESR) and no clinically significant decrease in albumin or hemoglobin.
*Exclusion criteria*
1. Subjects who were pregnant or breastfeeding.
2. Subjects who were experiencing an exacerbation at the time of screening or up to the time of the first dose.
3. Subjects who had undergone previous surgery for resection of bowel. (Subjects who had had surgery for perianal abscess more than 6 mo before dosing remained eligible.)
4. Subjects who had active fistulation.
5. Subjects who had a significant change in their immune-modulating maintenance medication in the 3 mo before screening and/or the start of dosing.
6. Subjects who had taken systemic steroids in the past 3 mo. Rectal and inhaled steroid use was permitted.
7. Subjects who were unable to take any oral feeding.
8. Subjects with feeding gastrostomies.
9. Subjects who had nonfood dietary supplementation changed for any reason within 1 mo before dosing.
10. Subjects who had received monoclonal antibodies in the 6 mo before dosing.
11. Subjects who had received antibiotics or probiotic dietary supplementation in the 2 wk before dosing. Subjects who had received foods with probiotics e.g., yoghurts were permitted to volunteer for the study.
12. Subjects who were receiving exclusive enteral feeding or had completed a course of exclusive enteral feeding in the 3 mo before dosing.
13. Subjects with concomitant autoimmune diseases (e.g., type 1 diabetes, juvenile arthritis, and psoriasis).
14. Female subjects of child-bearing potential unwilling to use effective contraception from the signing of the informed consent form until completion of 2 periods after the last dose. An effective method of birth control was defined as one which resulted in a low failure rate (i.e., less than 1% per year) when used consistently and correctly such as sterilization, implants, injectable, combined oral contraceptives, intrauterine device, condoms, and occlusive caps (cervical/vault caps) with spermicidal foam/gel/film/cream/pessary. True sexual abstinence was acceptable when this was in line with the preferred and usual lifestyle of the subject (periodic abstinence e.g., calendar, ovulation, symptothermal, postovulation methods, declaration of abstinence for the duration of the trial, and withdrawal were not acceptable methods of contraception).
15. Subjects with clinically significant, in the opinion of the investigator, elevations in platelets, white cell count, CRP, ESR, low albumin, or hemoglobin.
16. Subjects who were positive for the following viruses: HIV and hepatitis B or hepatitis C.
17. Subjects who smoked cigarettes or used other tobacco or nicotine containing produces, including e-cigarettes.
18. Subjects who had known sensitivity to any of the constituents of the investigational medicinal compound.
19. Diastolic blood pressure <50 or >90 mm Hg, a systolic blood pressure <100 or >150 mm Hg, a pulse <40 or >100 beats per minute (bpm) after resting for 5 min.
20. Subjects with clinically significantly abnormal ECGs or structural cardiac abnormalities e.g., valvular heart disease, patent foramen ovale.
21. Body mass index *Z* score less than −2.6 or greater than 2.6 (0 ± 2.6); i.e., below the second centile or greater than the ninety-eighth centile.
22. Any condition that, in the opinion of the investigator, might interfere with the primary study objective.
23. Subjects allergic to metronidazole and co-amoxiclav.
Exclusion criteria amended 18/12/16 to facilitate recruitment as follows:
3. Subjects who had undergone surgery for resection of bowel in the past 12 mo, or subjects who had undergone resection of bowel more than 12 mo ago, and subjects who had experienced an exacerbation in the past 12 mo or developed fistulae. (Subjects who had resection of bowel more than 12 mo ago, with no further requirement for surgery within the past 12 mo, with stable medications as per other inclusion/exclusion criteria and subjects who had surgery for perianal abscess more than 6 mo before dosing remained eligible.)
10. Subjects who were receiving a dose of monoclonal antibodies that had required adjustment because of clinical indications relating to their underlying CD and therefore had not been within a stable dosing range (e.g., 5 mg/kg 8 weekly, allowing for changes in subject weight) for the past 12 mo or who had evidence of an exacerbation. (Note: subjects who had missed or delayed occasional doses of monoclonal antibodies due to intercurrent illness, unavailability, examinations, holidays, other extraneous, or nonclinical reasons during the 12 mo remain eligible, providing the overall dosing regimen would have provided stable longer-term circulating drug levels in the opinion of the investigator.)

Thetanix is formulated as off-white, intrinsically enteric, size 0 capsules containing lyophilized *B. thetaiotaomicron*. Each capsule contains 10^7.73±1.43^ colony forming units (CFUs) and microcrystalline cellulose. Capsules were refrigerated until use. A single dose of Thetanix consisted of 3 capsules, comprising 10^8.2±1.4^ CFUs of *B. thetaiotaomicron*. The placebo capsules contained microcrystalline cellulose, but no *B. thetaiotaomicron* and were equivalent in size, weight, and appearance to the test formulation. Randomization was performed by an independent statistician on a 4 active: 1 placebo basis for both parts of the study. The allocation of kits to participants was performed centrally using an interactive voice/web response allocation system.

The occurrence of AEs was sought by questioning of the subject during dosing admissions and by completion of a diary card by subjects after discharge. AEs were checked at follow-up visits. AEs could have been detected when volunteered by the subject or through physical examination, laboratory test, or other assessments. All AEs were recorded in the case report form.

Traditional pharmacokinetic and pharmacodynamic assessments were not possible as part of this study because of the nature of the IMP. Disease activity was defined using physician's global assessment (PGA) and the weighted pediatric CD activity index (wPCDAI) (19). All recruits provided written informed consent for each part of the study.

### Part A: single dose

Part A (see Supplementary Table 1, Supplementary Digital Content 1, http://links.lww.com/AJG/B800) consisted of a screening visit, within 28 days before dosing, when a check was made of the subject's ability and willingness to swallow 3 empty size 0 placebo capsules. Three research visits were then required: for single-dose treatment on day 0 (visit 1) and post-treatment visits on day 1 (visit 2) and day 7 (visit 3).

Subjects were requested to have a light breakfast 2 hours before attending the clinic. Subjects received a single oral dose (3 capsules) of Thetanix or placebo. No food was allowed for 4 hours after dosing, although water was freely available. This step was undertaken to normalize dosing conditions across the study.

Subjects were assessed (vital signs and AEs) at 2, 4, and 8 hours postdosing and then discharged. Subjects completed electronic diaries including the recording of body temperature orally twice daily for 7 days alongside any side effects. Body temperatures were taken on electronic thermometers provided by the sponsor.

Subjects were instructed to contact the research site if they developed a fever (2 body temperature recordings ≥38.0°C in 12 h or a single temperature ≥38.5°C) to arrange an unscheduled assessment and blood cultures.

A baseline stool was collected in the 72 hours before day 0, and a post-treatment stool from 48 hours after dosing. Stool samples were collected in part A as a proof of principle for the method deployed in part B.

All clinical and laboratory data from part A was reviewed by the SRC before proceeding to part B.

### Part B: multiple doses

After approval from the SRC, part B recruitment commenced (Table 1). Subjects who participated in part A were also allowed to participate in part B if they wished, after rescreening and reconsenting, for pragmatic recruitment purposes. For subjects that did not participate in the single-dose phase, a check was made of the subject's ability and willingness to swallow 3 empty size 0 placebo capsules at screening.

**Table 1.**
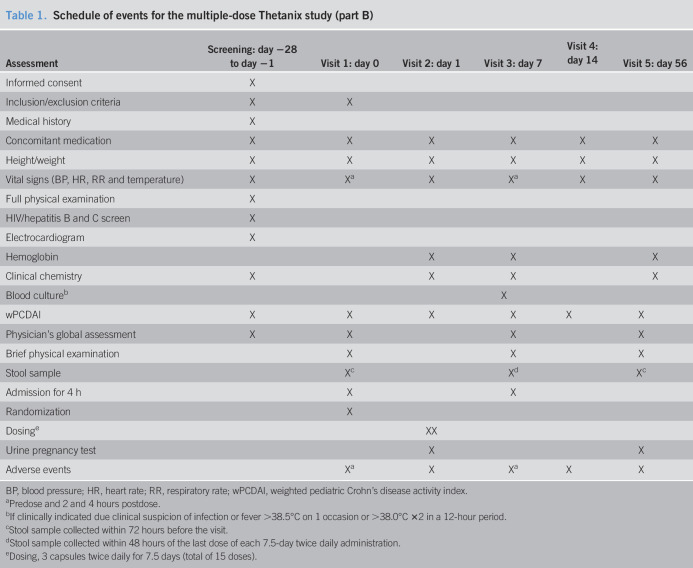
Schedule of events for the multiple-dose Thetanix study (part B)

Assessment	Screening: day −28 to day −1	Visit 1: day 0	Visit 2: day 1	Visit 3: day 7	Visit 4: day 14		Visit 5: day 56
Informed consent	X						
Inclusion/exclusion criteria	X	X					
Medical history	X						
Concomitant medication	X	X	X	X	X		X
Height/weight	X	X	X	X	X		X
Vital signs (BP, HR, RR and temperature)	X	X^a^	X	X^a^	X		X
Full physical examination	X						
HIV/hepatitis B and C screen	X						
Electrocardiogram	X						
Hemoglobin			X	X			X
Clinical chemistry	X		X	X			X
Blood culture^b^		X					
wPCDAI	X	X	X	X	X	X	
Physician's global assessment	X	X		X		X	
Brief physical examination		X		X		X	
Stool sample		X^c^		X^d^		X^c^	
Admission for 4 h		X		X			
Randomization		X					
Dosing^e^		XX					
Urine pregnancy test			X			X	
Adverse events		X^a^	X	X^a^	X	X	

BP, blood pressure; HR, heart rate; RR, respiratory rate; wPCDAI, weighted pediatric Crohn's disease activity index.

a Predose and 2 and 4 hours postdose.

b If clinically indicated due clinical suspicion of infection or fever >38.5°C on 1 occasion or >38.0°C ×2 in a 12-hour period.

c Stool sample collected within 72 hours before the visit.

d Stool sample collected within 48 hours of the last dose of each 7.5-day twice daily administration.

e Dosing, 3 capsules twice daily for 7.5 days (total of 15 doses).

Part B consisted of a screening visit, within 28 days before dosing and 5 research visits to commence treatment on day 0 (visit 1), a safety check 24 hours later on day 1 (visit 2), visits 7 and 14 days postday 0 (visits 3 and 4), and a final follow-up at day 56 (visit 5).

Part B treatment consisted of a twice daily dosing period of Thetanix or placebo (an hour before food) every 12 hours for 7.5 days. The first dose was taken in the clinic; the next 13 doses were taken at home, and the 15th dose was also taken in the clinic. The first and last doses were observed in the research facility, each with a 4-hour stay afterward, with blood pressure and temperature checks at 2- and 4-hours postdosing.

Subjects completed electronic diaries at home for 14 days after commencing treatment on day 0 (visit 1). The diaries collected the same information as in part A and subjects also recorded the times they took their study medication. Subjects were given access to electronic diaries in which to record body temperature twice daily, this time for 14 days, alongside any side-effects experienced and instructed to contact the research site if they developed a fever to arrange an unscheduled assessment and blood cultures.

Clinical disease activity indices were obtained on up to 6 occasions and blood sampling was undertaken on 4 (see Supplementary Table 1, Supplementary Digital Content 1, http://links.lww.com/AJG/B800 and Table 1). Vital signs and a medication check were undertaken during all visits. Three stool samples were requested—a baseline stool in the 72 hours before day 0, a post-treatment stool from the 48 hours after day 7, and a follow-up stool in the in the 72 hours before day 56.

The following parameters were evaluated over the course of the study: vital signs, height, weight, and calculation of body mass index *z* scores; calculation of PGA and wPCDAI scores; laboratory findings, fecal calprotectin (part B only), and stool microbiota.

### Laboratory

Routine hematology and serum biochemistry testing (full blood count, erythrocyte sedimentation rate, urea and electrolytes, liver function tests, bone profile, and C-reactive protein) was undertaken within accredited hospital laboratories linked to the research sites where subjects were recruited.

Subjects were provided with stool collection kits and instructions and asked to send samples to a central laboratory. Each stool sample was split in 2 by participants before posting. One sample was placed into a plain universal container for calprotectin analysis and another into the stool collection tube complete with stabilization buffer produced by Invitek Molecular GmbH (Berlin, Germany) for microbiome work.

### Statistical analysis

Analysis of clinical variables was undertaken in Microsoft Excel 2019 and IBM SPSS Statistics Version 26. Analysis of microbiome data is described in Supplementary Methods (see Supplementary Digital Content 1, http://links.lww.com/AJG/B800).

### Ethical considerations

Ethics committee approval was received for the study (Ref: 15/WS/0166). The study was registered on ClinicalTrials.gov (Ref: NCT02704728) and EduraCT (Ref: 2014-005666-29). Subjects were paid £100 for participation in part A and £200 for part B as agreed by the ethics review committee.

Details on fecal calprotectin, microbiome, and real-time polymerase chain reaction (RT-PCR) analyses are provided in Supplementary Methods (see Supplementary Digital Content 1, http://links.lww.com/AJG/B800).

## RESULTS

In total, 23 subjects were screened for this study—10 in part A and 13 in part B. Eight completed part A (6 active and 2 placebo) and 10 completed part B (8 active and 2 placebo; Table 2). One subject was excluded from part A because of nonavailability of the IMP and the other failed screening. Three screened subjects were excluded from part B—one failed screening, one attended screening but cancelled their dosing visit, and one withdrew consent after screening.

**Table 2.**
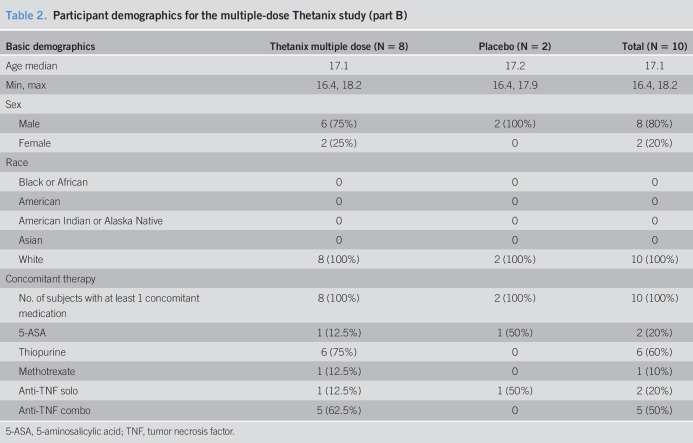
Participant demographics for the multiple-dose Thetanix study (part B)

Basic demographics	Thetanix multiple dose (N = 8)	Placebo (N = 2)	Total (N = 10)
Age median	17.1	17.2	17.1
Min, max	16.4, 18.2	16.4, 17.9	16.4, 18.2
Sex			
Male	6 (75%)	2 (100%)	8 (80%)
Female	2 (25%)	0	2 (20%)
Race			
Black or African	0	0	0
American	0	0	0
American Indian or Alaska Native	0	0	0
Asian	0	0	0
White	8 (100%)	2 (100%)	10 (100%)
Concomitant therapy			
No. of subjects with at least 1 concomitant medication	8 (100%)	2 (100%)	10 (100%)
5-ASA	1 (12.5%)	1 (50%)	2 (20%)
Thiopurine	6 (75%)	0	6 (60%)
Methotrexate	1 (12.5%)	0	1 (10%)
Anti-TNF solo	1 (12.5%)	1 (50%)	2 (20%)
Anti-TNF combo	5 (62.5%)	0	5 (50%)

5-ASA, 5-aminosalicylic acid; TNF, tumor necrosis factor.

Recruits to part A were 75% male patients with a median age of 17.1 years at randomization (range 16.1–18.2 years), whereas those in part B were 80% male patients with a median age of 17.1 years (range 16.4–18.2 years). Three participants completed both part A and part B.

All participants were on concomitant medication. Immunomodulators were prescribed in 75% of part A participants (66.7% active and 100% placebo) and anti-TNFα in 37.5% of part A (33.3% active and 50% placebo). Concomitant IBD therapy in part B is outlined in Table 2.

Treatment compliance was not measured in part A because the IMP was administered under direct supervision. Reported treatment compliance in part B was 99%, with one subject on active Thetanix reporting 14/15 doses and all others reporting full compliance.

### Safety

A summary of all AEs is reported in Table 3. No serious AEs or deaths occurred within the study. Two Thetanix recipients in part A reported 4 AEs in total (abdominal pain, eructation, flatulence, and gastroesophageal reflux). The eructation, flatulence, and gastroesophageal reflux in one participant were deemed treatment related by an investigator. Neither of the part A placebo recipients reported any AEs. All PGA scores were “quiescent” in part A. wPCDAI scores in part A ranged from 0 to 20. All scores were in remission apart from one placebo-treated recruit who scored 20 at the baseline visit, but not at screening (Table 4).

**Table 3.**
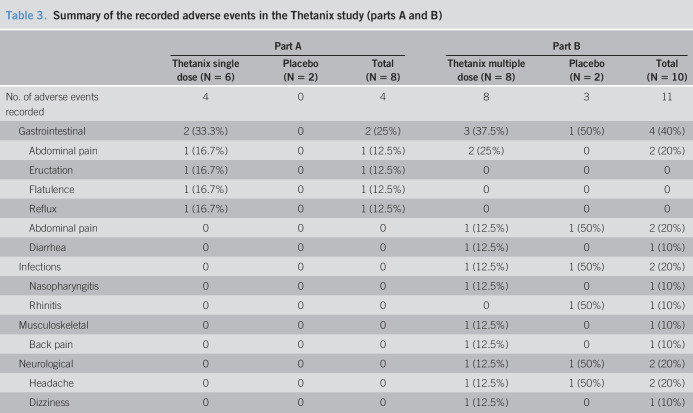
Summary of the recorded adverse events in the Thetanix study (parts A and B)

	Part A			Part B		
	Thetanix single dose (N = 6)	Placebo (N = 2)	Total (N = 8)	Thetanix multiple dose (N = 8)	Placebo (N = 2)	Total (N = 10)
No. of adverse events recorded	4	0	4	8	3	11
Gastrointestinal	2 (33.3%)	0	2 (25%)	3 (37.5%)	1 (50%)	4 (40%)
Abdominal pain	1 (16.7%)	0	1 (12.5%)	2 (25%)	0	2 (20%)
Eructation	1 (16.7%)	0	1 (12.5%)	0	0	0
Flatulence	1 (16.7%)	0	1 (12.5%)	0	0	0
Reflux	1 (16.7%)	0	1 (12.5%)	0	0	0
Abdominal pain	0	0	0	1 (12.5%)	1 (50%)	2 (20%)
Diarrhea	0	0	0	1 (12.5%)	0	1 (10%)
Infections	0	0	0	1 (12.5%)	1 (50%)	2 (20%)
Nasopharyngitis	0	0	0	1 (12.5%)	0	1 (10%)
Rhinitis	0	0	0	0	1 (50%)	1 (10%)
Musculoskeletal	0	0	0	1 (12.5%)	0	1 (10%)
Back pain	0	0	0	1 (12.5%)	0	1 (10%)
Neurological	0	0	0	1 (12.5%)	1 (50%)	2 (20%)
Headache	0	0	0	1 (12.5%)	1 (50%)	2 (20%)
Dizziness	0	0	0	1 (12.5%)	0	1 (10%)

**Table 4.**
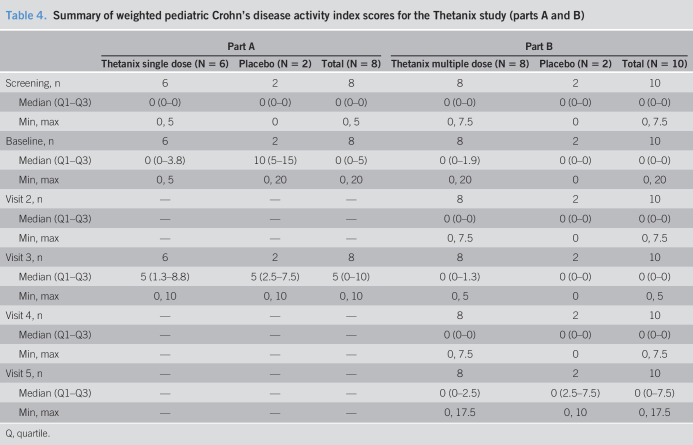
Summary of weighted pediatric Crohn's disease activity index scores for the Thetanix study (parts A and B)

	Part A			Part B		
	Thetanix single dose (N = 6)	Placebo (N = 2)	Total (N = 8)	Thetanix multiple dose (N = 8)	Placebo (N = 2)	Total (N = 10)
Screening, n	6	2	8	8	2	10
Median (Q1–Q3)	0 (0–0)	0 (0–0)	0 (0–0)	0 (0–0)	0 (0–0)	0 (0–0)
Min, max	0, 5	0	0, 5	0, 7.5	0	0, 7.5
Baseline, n	6	2	8	8	2	10
Median (Q1–Q3)	0 (0–3.8)	10 (5–15)	0 (0–5)	0 (0–1.9)	0 (0–0)	0 (0–0)
Min, max	0, 5	0, 20	0, 20	0, 20	0	0, 20
Visit 2, n	—	—	—	8	2	10
Median (Q1–Q3)	—	—	—	0 (0–0)	0 (0–0)	0 (0–0)
Min, max	—	—	—	0, 7.5	0	0, 7.5
Visit 3, n	6	2	8	8	2	10
Median (Q1–Q3)	5 (1.3–8.8)	5 (2.5–7.5)	5 (0–10)	0 (0–1.3)	0 (0–0)	0 (0–0)
Min, max	0, 10	0, 10	0, 10	0, 5	0	0, 5
Visit 4, n	—	—	—	8	2	10
Median (Q1–Q3)	—	—	—	0 (0–0)	0 (0–0)	0 (0–0)
Min, max	—	—	—	0, 7.5	0	0, 7.5
Visit 5, n	—	—	—	8	2	10
Median (Q1–Q3)	—	—	—	0 (0–2.5)	0 (2.5–7.5)	0 (0–7.5)
Min, max	—	—	—	0, 17.5	0, 10	0, 17.5

Q, quartile.

Four active recipients in part B reported 8 AEs in total (3 instances of abdominal pain, one each of back pain, diarrhea, nasopharyngitis, dizziness, and headache). The dizziness, abdominal pain, and headache in one participant were deemed treatment related by an investigator. One placebo recipient in part B reported rhinitis, headache, and abdominal pain, which was deemed unrelated.

All pregnancy tests undertaken were negative. All HIV and hepatitis screens were negative. There were no clinically meaningful changes in vital signs, ECG recordings, or hematology/biochemistry results during the study. Importantly, no subject had a raised temperature during the study, and hence, no blood cultures and no antibiotic courses were required.

All PGA scores in part B were marked “quiescent” or “mild.” One subject had a rating of “mild” at baseline, but not at screening nor at day 56 (visit 5), whereas one had a baseline assessment of “quiescent” and was marked “mild” at day 56. All other assessments were “quiescent.” Full wPCDAI data are presented in Table 4; however, scores ranged from 0 to 20 in part B, with all scores achieving remission (<12.5) throughout the study with the exception of one baseline score of 20, in a participant with a remission score at screening, and one day 56 (visit 5) score of 17.5, both in the active group.

Thirteen of 16 (81%) possible stool samples from part A and 29/30 (97%) stools from part B were received. The 4 missing stool samples comprised one each from part A active day 0, part A active day 1, part A placebo day 0, and part B active day 56.

There was no significant change in median calprotectin values across 5 visits in part B—87.8 (range 4.4–447) to 50.5 (range 5.3–572), *P* = 1.00 by the Fisher exact test in the active group and 64.6 (range 14.9–114.3) to 123.5 (range 8.2–238.8), *P* = 1.00 in the placebo group. Calprotectin summary data are presented in Table 5 and Supplementary Figure 1 (see Supplementary Digital Content 1, http://links.lww.com/AJG/B800).

**Table 5.**
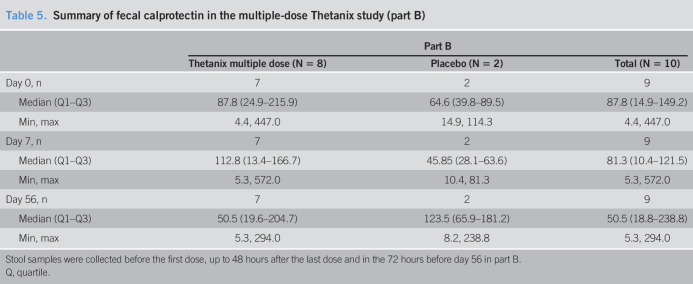
Summary of fecal calprotectin in the multiple-dose Thetanix study (part B)

	Part B		
	Thetanix multiple dose (N = 8)	Placebo (N = 2)	Total (N = 10)
Day 0, n	7	2	9
Median (Q1–Q3)	87.8 (24.9–215.9)	64.6 (39.8–89.5)	87.8 (14.9–149.2)
Min, max	4.4, 447.0	14.9, 114.3	4.4, 447.0
Day 7, n	7	2	9
Median (Q1–Q3)	112.8 (13.4–166.7)	45.85 (28.1–63.6)	81.3 (10.4–121.5)
Min, max	5.3, 572.0	10.4, 81.3	5.3, 572.0
Day 56, n	7	2	9
Median (Q1–Q3)	50.5 (19.6–204.7)	123.5 (65.9–181.2)	50.5 (18.8–238.8)
Min, max	5.3, 294.0	8.2, 238.8	5.3, 294.0

Stool samples were collected before the first dose, up to 48 hours after the last dose and in the 72 hours before day 56 in part B.

Q, quartile.

**Figure 1.**
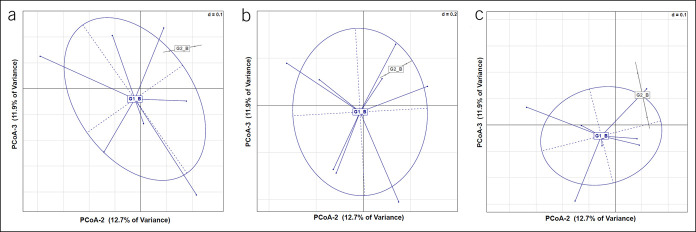
Microbiota profiles of treatment groups in the multiple-dose Thetanix study (part B) on days 0, 7, and 56 based on Bray-Curtis Dissimilarities. (**a**) Day 0 (n = 10), (**b**) day 7 (n = 10), and (**c**) day 56 (n = 9). G1, active treatment; G2, placebo; PCoA, principle components analysis. No significant differences were found in microbiota profiles between the groups at day 0 (*P* = 0.09), day 7 (*P* = 0.589), or day 56 (*P* = 0.299) in part B.

All 42 stool samples were sequenced for microbiome analysis. 81,672 ± 12,704 reads were generated per sample. Microbiome analysis demonstrated no significant differences between active and placebo groups by Bray-Curtis dissimilarity in part B at days 0, 7, and 56 (Figure 1a–c).

**Figure 2.**
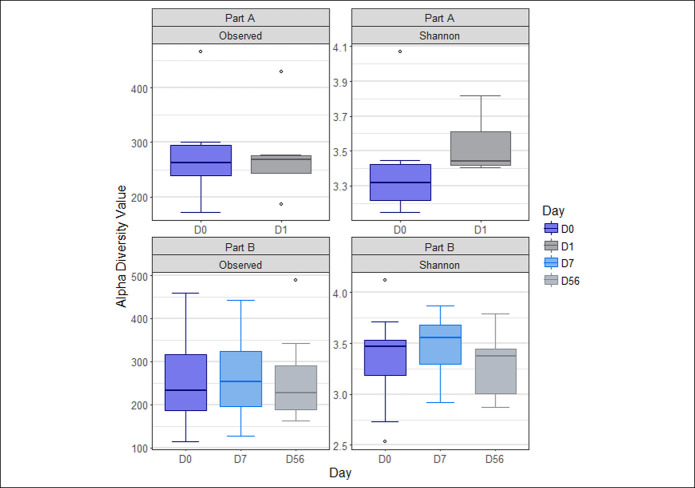
Effect of Thetanix on microbiota diversity in part A and part B using observed species and Shannon Metrics.

Part A allowed exploration of diversity changes in the active treatment group across a shorter time period (day 0 to day 1) than part B (day 0 to day 7 and day 56) (Figure 2). There was no significant difference in microbiota diversity for the active group in part A (observed *P* value = 0.236, Shannon *P* value = 0.140) from day 0 to day 1. Shannon diversity was however found to be significantly different (observed *P* value = 0.436, Shannon *P* value = 0.023) across the study time points in part B with an increase in diversity from day 0 to day 7 which decreased at day 56.

Microbiota evenness was also explored in the active group by the Shannon diversity index (see Supplementary Figure 2, Supplementary Digital Content 1, http://links.lww.com/AJG/B800). A nonsignificant increase in microbiota evenness was observed across the time points in part A (day 0 to day 1, *P* = 0.07), and a significant difference was observed in part B (*P* = 0.03) across the study time points with an increase in evenness from day 0 to day 7, which decreased again at day 56.

No significant differences were seen in temporal microbiome stability in part A or part B active or placebo groups (data not shown). No significant differences were seen in taxa abundance or functional pathway abundance across part B (data not shown).

All 42 stool samples and 2 spike-in positive control samples were screened for *B. thetaiotaomicron* by RT-PCR (see Supplementary Table 2, Supplementary Digital Content 1, http://links.lww.com/AJG/B800). *B. thetaiotaomicron* was identified in the positive controls but was only identified in 5 (11.9%) samples; hence, further analysis of these data was not possible. RT-PCR was positive for *B. thetaiotaomicron* in one placebo recipient in part A.

## DISCUSSION

Our study provides evidence of the tolerability and safety of *B. thetaiotaomicron*. Reported compliance with therapy was high, there were no serious AEs, and the 2 participants who suffered treatment-associated AEs had minor, self-limiting symptoms that did not require specific treatment nor cessation of the trial. In these patients with CD in remission, there was no increase in wPCDAI or PGA during the study, and there was no significant change in calprotectin levels in part B.

This first-in-humans phase I study of the novel live biotherapeutic (next-generation probiotic) *B. thetaiotaomicron* represents a rarity within the medical literature. We have traditionally considered 4 phases of drug-based research in humans—from phase I safety to phase IV postmarketing surveillance (Table 6)—but previous clinical studies of probiotics in IBD have generally not followed the same path, with reported studies often being more associated with phase II or phase III than the exploration of safety. Importantly, our study was deliberately small and was designed as a preliminary investigation of safety and tolerability in a young population with known disease. It was therefore underpowered to explore dosing, efficacy, or mechanisms. Exploration of these factors will be a fundamental component of follow-on phase II studies and beyond. As we explore more novel agents in distinct settings, particularly as we seek to look at the biological utility of an organism to provide or supplement a function rather than simply to colonize a niche, there is a need to revisit our conventional approach to the testing of drug-based therapies and to adjust this for the study of novel microbial agents. There are significant and specific issues related to the study of live organisms as therapy that are not easily mapped to the traditional phases of drug-based research (expanded in Table 6), most notably in the assessment of their pharmacokinetic and pharmacodynamic properties. This has an important impact on the exploration of both dosing and the monitoring of therapeutic “levels” of the organism in question. Specifically, we cannot prove nonefficacy of a live biotherapeutic if we cannot determine adequate dosing or adequate uptake of the organism in the appropriate niche. The converse is of course not true; hence, in situations where clinical efficacy is proven, these aspects can be assumed, but there is a danger of discarding live biotherapeutics for nonefficacy without first addressing these fundamental components.

**Table 6.**
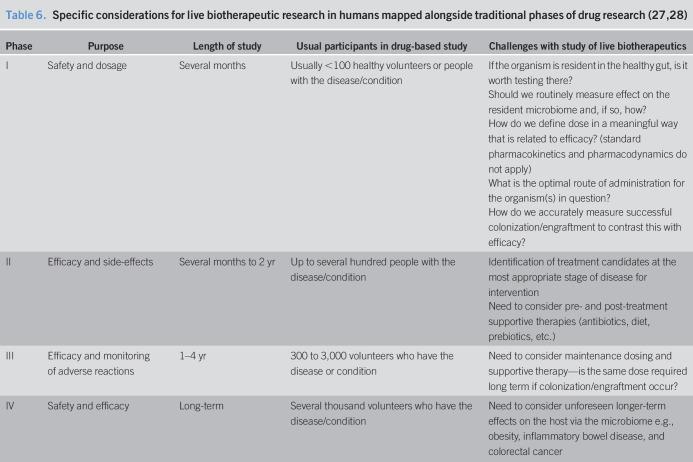
Specific considerations for live biotherapeutic research in humans mapped alongside traditional phases of drug research (27,28)

Phase	Purpose	Length of study	Usual participants in drug-based study	Challenges with study of live biotherapeutics
I	Safety and dosage	Several months	Usually <100 healthy volunteers or people with the disease/condition	If the organism is resident in the healthy gut, is it worth testing there?
				Should we routinely measure effect on the resident microbiome and, if so, how?
				How do we define dose in a meaningful way that is related to efficacy? (standard pharmacokinetics and pharmacodynamics do not apply)
				What is the optimal route of administration for the organism(s) in question?
				How do we accurately measure successful colonization/engraftment to contrast this with efficacy?
II	Efficacy and side-effects	Several months to 2 yr	Up to several hundred people with the disease/condition	Identification of treatment candidates at the most appropriate stage of disease for intervention
				Need to consider pre- and post-treatment supportive therapies (antibiotics, diet, prebiotics, etc.)
III	Efficacy and monitoring of adverse reactions	1–4 yr	300 to 3,000 volunteers who have the disease or condition	Need to consider maintenance dosing and supportive therapy—is the same dose required long term if colonization/engraftment occur?
IV	Safety and efficacy	Long-term	Several thousand volunteers who have the disease/condition	Need to consider unforeseen longer-term effects on the host via the microbiome e.g., obesity, inflammatory bowel disease, and colorectal cancer

Our study of the impact of Thetanix on the human fecal microbiome was largely exploratory; the return rate of stool samples was high (81% and 97%), and there was no significant impact on overall microbial composition, temporal microbiome stability, taxa abundance, or functional pathway abundance during the study. The significant changes in Shannon diversity and evenness in the active arm of part B are preliminary and need replication in a larger phase II efficacy study to better understand their relevance for a broader mechanism of action.

Our poor pick-up rate of *B*. *thetaiotaomicron* in fecal samples by RT-PCR warrants discussion—clearly, this was a first attempt at demonstrating positive colonization by a noninvasive, reproducible, and relatively inexpensive laboratory assay. We have explored the efficacy of this assay in the laboratory setting and demonstrated high sensitivity for pick-up of the same strain of *B. thetaiotaomicron* including with spiked samples of fecal DNA from this study. We are therefore confident that this result is a true negative to the limits of the test because we did detect the organism in some samples, and so, it was not entirely absent. There are several possibilities that could explain this observation—transit of the organism is not reflected in our stool collection timing, so we are missing it; our dose is insufficient for fecal excretion and may need to be higher or more frequent; participants did not take the therapy despite reporting compliance (which seems unlikely, given the pervasiveness of low pick-up); the organism is being degraded by gastric acidity or during transit through the gut; or all the administered *B. thetaiotaomicron* is colonizing and so not being excreted. Studying the latter would require before/after biopsies of the colon that would not be ethically acceptable in children and would be hard to justify in an adult study in the absence of monitoring active disease. Stool samples are at best a surrogate for measuring colonization with probiotics, and hence, poor detection in this medium does not infer no colonization. Follow-on studies should specifically explore dosing and whether there is a dosing threshold above which *B. thetaiotaomicron* is detectable in stool. A potential alternative approach for exploration of colonization in follow-on studies would be evaluation of the mechanistic impact of the organism on fecal metabolomics and markers of gut inflammation such as cytokine levels, particularly TNFα. All blood sampling and analysis in this study was undertaken in hospital laboratories local to recruitment, and hence, serum TNFα monitoring was not possible in this early phase study. This would be an interesting biomarker to explore in follow-on research.

On the subject of dosing, *B. thetaiotaomicron* is a symbiont in the bowel of healthy humans and can be detected in stool at an average concentration of 1.39 × 10^8^ CFUs/g of feces (20). The median passage of stool is 128 g/person/day (21); hence, a healthy individual sheds a median 1.78 × 10^10^ *B. thetaiotaomicron* each day. It has also been estimated that 6% of all bacteria in the healthy human intestine are *B. thetaiotaomicron* (22), which would equate to ∼6 × 10^13^ organisms. Limited data exist in the literature on the prevalence of *B. thetaiotaomicron* in the gut of patients with CD, but it does not seem to be abundant (14,23,24). Importantly, however, Thetanix does not aspire to replace an absent nor increase levels of a low-prevalence organism, it is instead intended to provide a live biotherapeutic source of a potentially beneficial anti-inflammatory mechanism, mitigating against the high levels of TNFα that have long been associated with CD inflammation (25,26). The main objective therefore was to dose to reduce inflammation in the gut by increasing *B. thetaiotaomicron*, and hence its effects on the NF-κB signaling pathways and subsequently TNFα. For this study, the dose administered was 3 capsules, each containing 10^7.73±1.43^ CFUs, therefore giving a total daily dose of 10^8.21±1.43^ CFUs. Twice daily dosing for 7.5 days (15 doses) in part B would therefore give a cumulative dose between 8.98 × 10^7^ and 6.5 × 10^10^ CFUs. The viable bacterial cell count is considered to be the key potency parameter for Thetanix because the critical measure of activity was considered applicable for therapeutic effect. The chosen dose for this study was, however, also pragmatically based on the maximum number of viable bacteria that could be loaded into a capsule, coupled with a realistic number of daily doses for the participants in the study that would be reproducible in clinical practice. Crossover was allowed between part A and part B in this study, with 3 subjects undergoing further randomization in part B. Crossover of a single-dose active product from part A into the placebo arm of part B was therefore possible within the study design. We did not control or analyze for this, given part B was single dose and crossover was not immediate. Future studies of Thetanix may need to explore higher doses or longer durations of treatment if subsequent efficacy evaluations are considered underdosed. As discussed above, ensuring adequate biotherapeutic delivery is a key challenge within this emerging field of therapeutic research.

*B. thetaiotaomicron* (as Thetanix) with twice daily doses over a 7.5-day dosing period is well tolerated by adolescents with CD who are in remission. It does not result in significant AEs or toxicity over this time period and does not detrimentally alter clinical or laboratory parameters including fecal calprotectin. Although exploratory, limited evidence points to a positive effect on microbial diversity and evenness though, on the whole, *B. thetaiotaomicron* had a limited impact on the host fecal microbiota during this initial study. Future studies should explore the optimum dose and duration of Thetanix and signals of its efficacy as an induction and maintenance agent in CD.

## CONFLICTS OF INTEREST

**Guarantor of the article:** Richard Hansen, PhD.

**Specific author contributions:** R.H., I.R.S., R.M., S.A., C.T., P.H., L.G., R.K.R., and D.C.W.: identified and recruited subjects for the study, completed follow-up visits, and populated case report forms. R.H., J.D.W., G.K., R.K.R., and D.C.W.: designed the study and agreed any required protocol changes. I.B.J., D.P.M., and E.A.O.H.: completed all laboratory experiments, laboratory data analysis, and bioinformatics. R.H.: was the principle investigator, undertook clinical statistical analyses, and wrote the first draft of the paper. All authors approved the final version of the manuscript.

**Financial support:** R. Hansen and R. K. Russell are supported by NHS Research Scotland Senior Researcher Fellowships. P. Henderson is supported by an NHS Research Scotland Career Research Fellowship.

**Potential competing interests:** 4D pharma sponsored the study and produce Thetanix. R. Hansen, R. K. Russell, and D. C. Wilson were paid consultancy fees during the design phase of the study. R. Hansen has received further consultancy fees regarding Phase II work and travel expenses for presenting this work at Digestive Disease Week. R. Muhammed has received travel expenses from 4D pharma. I. B. Jeffery, D. P. Mullins, E. A. O'Herlihy, and J. D. Weinberg are employees of 4D pharma. G. Kitson is an employee of ProPharma Partners Limited and was a paid consultant of 4D pharma.

**Clinical trial registration:** ClinicalTrials.gov (Ref: NCT02704728) and EduraCT (Ref: 2014-005666-29).

**Study Highlights**

**WHAT IS KNOWN**

✓ The microbiome is important in Crohn's disease pathogenesis.
✓ Probiotics have been disappointing as therapy for Crohn's disease to date, but most organisms studied have been selected for convenience, availability, or ease of manufacture.
✓ *Bacteroides thetaiotaomicron* attenuates gut inflammation via antagonism of transcription factor NF-κB and subsequent reduction of TNFα.

**WHAT IS NEW HERE**

✓ *B. thetaiotaomicron* is a good candidate for translation to a novel therapy as a potentially mucosally active anti-TNFα.
✓ *B. thetaiotaomicron* as Thetanix is well tolerated with a seemingly good safety profile.
✓ wPCDAI and calprotectin were unaffected by Thetanix dosing, but bacterial diversity seemed to increase in treated subjects.

## Supplementary Material

SUPPLEMENTARY MATERIAL
