# Supplementary material for: A Double-Blind, Placebo-Controlled Trial to Assess Safety and Tolerability of (Thetanix) Bacteroides thetaiotaomicron in Adolescent Crohn's Disease
Source: Clin Transl Gastroenterol. 2020 Dec 18;12(1):e00287. doi: 10.14309/ctg.0000000000000287 (PMC7752678; doi:10.14309/ctg.0000000000000287)
Supplement: SUPPLEMENTARY MATERIAL [file ct9-12-e00287-s002.docx]

**Supplementary**

Supplementary Table 1: Schedule of Events for Single Dose Thetanix® Study (Part A)

| **Assessment** | **Screening Day -28 to Day -1** |  |  |  |
| --- | --- | --- | --- | --- |
|  |  | **Visit 1**  **Day 0** | **Visit 2**  **Day 1** | **Visit 3 Day**  **7** |
|  |  |  |  |  |
| Informed consent | X |  |  |  |
| Inclusion/Exclusion criteria | X | X |  |  |
| Medical history | X |  |  |  |
| Concomitant medication | X | X | X | X |
| Height/weight | X | X |  |  |
| Vital signs (BP, HR, RR and temperature) | X | X^a^ | X | X |
| Full physical examination | X |  |  |  |
| HIV/Hepatitis B and C screen | X |  |  |  |
| Electrocardiogram | X |  |  |  |
| Hemoglobin | X |  | X | X |
| Clinical chemistry | X |  | X | X |
| Blood culture^d^ |  | X | | |
| wPCDAI | X | X |  | X |
| Physician’s global assessment | X | X |  | X |
| Brief physical examination |  | X |  | X |
| Stool sample |  | X^b^ | X^c^ |  |
| Admission for 8 hours |  | X |  |  |
| Randomization |  | X |  |  |
| Dosing |  | X |  |  |
| Urine pregnancy test |  | X |  |  |
| Adverse events |  | X^a^ |  |  |

BP=blood pressure, HR=heart rate, RR=respiratory rate, HIV=human immunodeficiency virus, wPCDAI=weighted pediatric Crohn’s disease activity index.
^a^ Predose, 2, 4, and 8 hours post‑dose.
^b^ Stool sample collected within 72 hours prior to Visit 1.
^c^ Stool sample collected within 48 hours post‑dosing.
^d^ If clinically indicated due clinical suspicion of infection or fever >38.5°C on 1 occasion or >38.0°C ×2 in a 12-hour period.


**Supplementary Table 2:** ***B. thetaiotaomicron* Quantification by RT-PCR in Thetanix® Study (Parts A and B)**

| **Treatment/**  **Subject number** | **Part A D0** | **Part A D1** | |
| --- | --- | --- | --- |
| **Thetanix** |  |  | |
| 201 | Not detected | Not detected | |
| 202 | Not detected | Not detected | |
| 105 | Not detected | Not detected | |
| 301 | Not detected | Not detected | |
| 401 | Not detected | Not detected | |
| **Placebo** |  |  | |
| 103 | 4.64×10^5^ CFUs | 1.47×10^5^ CFUs | |
| 402 | No sample | Not detected | |
|  | **Part B D0** | **Part B D7** | **Part B D56** |
| **Thetanix** |  |  | |
| 152 | Not detected | Not detected | Not detected |
| 352 | Not detected | Not detected | No sample |
| 451 | Not detected | 7.49×10^4^ CFUs | 4.99×10^4^ CFUs |
| 452 | Not detected | Not detected | Not detected |
| 453 | Not detected | 3.06×10^1^ CFUs | Not detected |
| 454 | No sample | No sample | No sample |
| 455 | Not detected | Not detected | Not detected |
| 551 | Not detected | Not detected | Not detected |
| 552 | Not detected | Not detected | Not detected |
| **Placebo** |  |  | |
| 153 | Not detected | Not detected | Not detected |
| 351 | Not detected | Not detected | Not detected |

**Supplementary Figure 1: Fecal Calprotectin Data From Multiple Dose Thetanix**® **Study (Part B)**

**
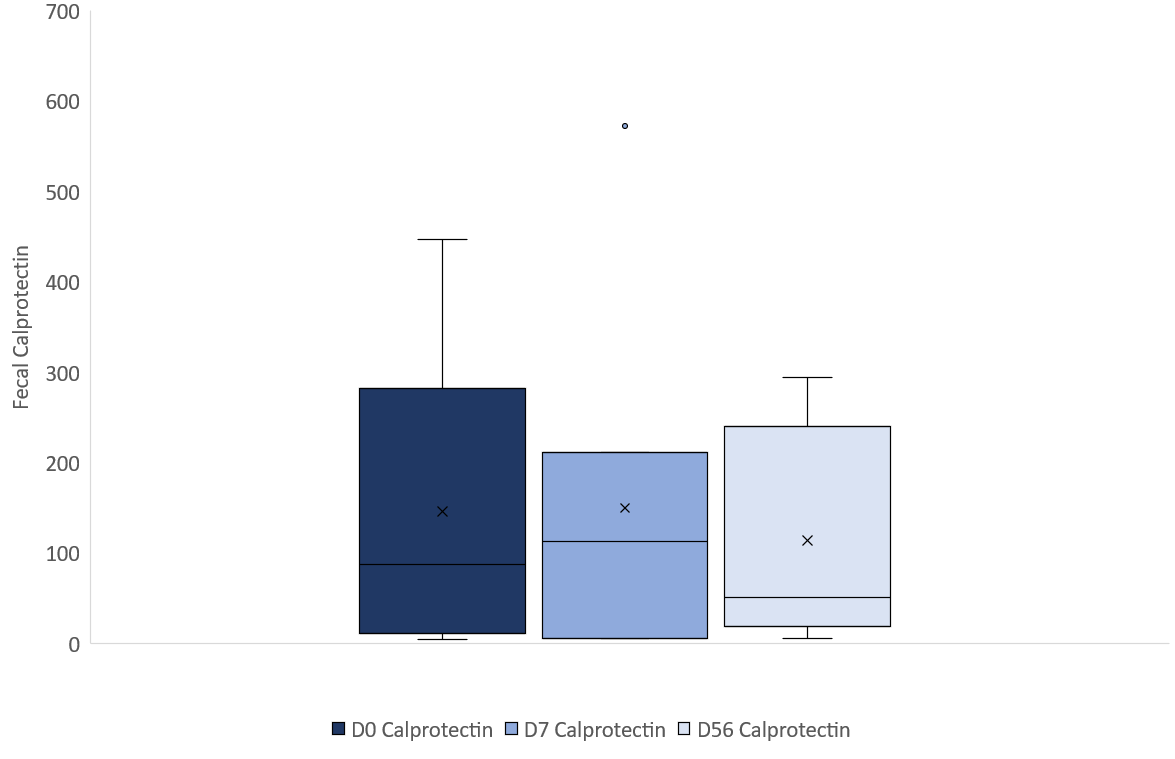
**

**Supplementary Figure 2: Microbiota Evenness by Shannon Diversity Index in patients receiving Thetanix**® **(Parts A and B).**


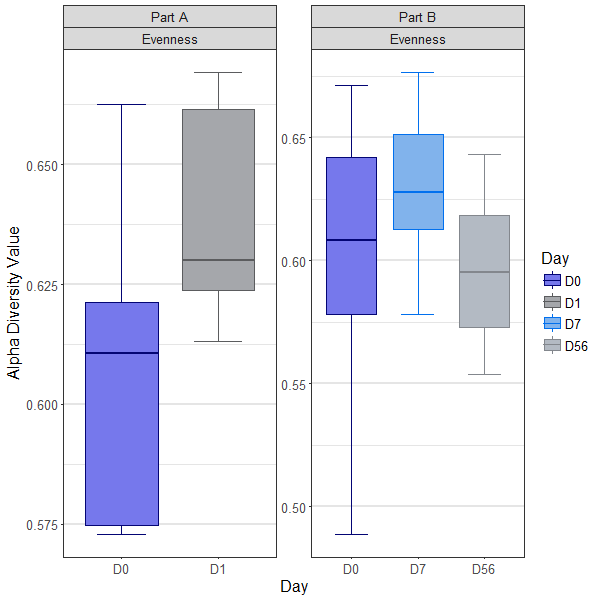


**Supplementary Methods**

Dosing Visit

During Day 0 subjects arrived in the morning approximately 2 hours after having consumed a light breakfast (e.g., cereal, fruit, or toast). Following eligibility confirmation, a review of medical history and brief physical examination and confirmation that subjects were not experiencing an exacerbation, baseline assessments were undertaken including: changes in medication, assessment of vital signs, a urine pregnancy test for all female subjects who were post-menarche, height, weight and calculation of body mass index (BMI) Z scores, recalculation of physician’s global assessments (PGA) and weighted pediatric Crohn’s disease activity index (wPCDAI) score^19^.

Fecal Calprotectin

Fecal samples received in universal containers without buffer were frozen on receipt then tested in batches in triplicate following the manufacturer’s guidelines for the CALPRO calprotectin enzyme-linked immunosorbent assay (ELISA) test alkaline phosphatase kit (CALPRO AS, Lysaker, Norway). Samples were diluted 1:50 for the ELISA assay, however sample results that were higher than the standard range were re-run at a lower dilution factor. Subject samples from different time points were run on the same assay plate. Quality controls were included in each run and quality parameters were assessed as per manufacturer’s guidelines. Fecal sample results were expressed as mg/kg.

Microbiome Data Analysis

DNA was extracted and amplified using 0.25g of fecal sample, taken from individual stool collection tubes with stabilization buffer, which were stored at room temperature. The 16S rRNA gene amplicon preparation method targeting the V3-V4 regions described by Brown *et al*^29^ was used with minor modifications. The bead beating tubes comprised of 4 x 3.5mm glass beads and 0.5g of 0.1mm zirconia beads. The samples were homogenized by bead beating for 3 x 60 second cycles and cooled on ice between every cycle. DNA was visualized on 0.8% agarose gels and quantified with a SimpliNano Spectrometer (Biochrom™, Massachusetts, USA). The following primers were used: Forward Primer (S-D-Bact-0341-b-S-17) = 5'

TCGTCGGCAGCGTCAGATGTGTATAAGAGACAGCCTACGGGNGGCWGCAG ; Reverse Primer (S-D-Bact-0785-a-A-21) = 5'

GTCTCGTGGGCTCGGAGATGTGTATAAGAGACAGGACTACHVGGGTATCTAATCC. The PCR master mix included 2X Phusion Taq High-Fidelity Mix (Thermo Scientific, Ireland) and 15ng of DNA. 16S rRNA gene amplicon sequencing was carried out by GATC Biotech, Germany on the Illumina MiSeq platform on a 2 × 250 bp paired end sequencing run.

Between sample variation (Beta-diversity) was investigated using the Bray-Curtis dissimilarity metric generated using the vegan library and visualized using principal coordinate analysis (PCoA) (ade4 library). Alpha diversity was investigated using observed species and the Shannon diversity index^30^ which represents number of taxa per sample (richness) and their relative abundances (evenness) within each sample respectively. Temporal stability is defined as the Bray-Curtis distance^31^ between time points for each sample. To establish whether there were significant differences in the global microbiome profiles between groups, Permutational multivariate analysis of variance (MANOVA) was performed on the dissimilarity matrix using the Adonis function in R. The Adonis tests were performed by group or timepoint. Boxplots were constructed using the ggplot2 package in the R statistical software. Boxplots provide a graphical representation of the median, quartiles, maximum and minimum of a dataset. Statistical significance was determined for diversity and stability metrics using mixed liner models with subject as a random variable. A negative binomial statistical methodology (DESeq2 algorithm) was used to identify taxonomic variables that were significantly differentially abundant within chosen comparisons. Raw p-values produced were adjusted for multiple testing using the Benjamini/Hochberg methodology^32^ where appropriate. Nominal p-values are reported for diversity and stability analyses. The PICRUSt (Phylogenetic Investigation of Communities by Reconstruction of Unobserved States) package^33^ was used to infer the functional-gene profile of a microbiome based on the 16S marker genes. Functional genes were predicted based on the KEGG Orthology classification^34^. Pathways that were differentially abundant between the treated groups and the vehicle were predicted using the functional-gene count profile matrix and the DESeq2 methodology. To filter out potentially spurious results, an adjusted p-value cut-off of 0.05 and an absolute value log2 fold change cut-off of 0.5 was used for the PICRUSt DESeq2 analysis only.

Statistical analysis was performed using R 3.4.0 statistical software.

*B. thetaiotaomicron* qPCR quantification

Sample preparation: Sample DNA extracts as described previously were diluted to 10 ng/µL and 40 ng/µL and used for *B. thetaiotaomicron* qPCR quantification.

The primers used for qPCR were designed by Diversigen (Texas, USA) with a target sequence partially coving the BT_2225 gene 73302-F: TCATCGGTGATAGCCTCTACCGCTC;73302-R: TTGCTACACGCACGGGTAGTGCCAA.

qPCR: Standards were prepared by amplifying the target sequencing of *B. thetaiotaomicron* (Thetanix® strain) using genomic DNA. The target amplicon qPCR product was quantified and then serially diluted ten-fold (in triplicate) to construct qPCR standard curves. The optimum annealing temperature for primers was assessed during assay optimization and an annealing temperature of 69°C was used. The qPCR conditions used were as detailed in FastStart Essential DNA Green Master Protocol version 6, Roche.

The qPCR contained 10 µL SYBR Green Master Mix 2×, 1 µL of each primer (10 µM), 5 µL template DNA and 3 µL molecular grade nuclease free water to make up a final reaction volume of 20 µL. DNA extracted from all samples was analyzed by qPCR using Roche LightCycler 96 system Instrument (Roche Applied Sciences, Indianapolis). Cycle threshold was measured during each amplification and target gene concentration was analyzed automatically by the absolute quantification method by the LightCycler Software (LightCycler® 96 SW 1.1). Standards and samples were run in triplicate. A positive control (known target sequence quantity 1×10^5^ CFUs) was included in triplicate during each qPCR. An additional reference standard was prepared from *B. thetaiotaomicron* MR×1233 g DNA extracted from enumerated *B. thetaiotaomicron* culture at 6×10^8^ CFU/mL using the DNeasy PowerLyzer Microbial Kit (Qiagen, Maryland, USA).

Subject samples were run in triplicate at 2 dilutions (10 ng/µL and 40 ng/µL). Subject samples from different time points were run on the same assay plate. An inoculum of 10^4^ to 10^5^ CFUs was spiked into two subject samples which were used as an in-house positive control to confirm detection of *B. thetaiotaomicron.* The copies of target amplicon (partial BT2225 gene) of *B. thetaiotaomicron* present in the sample were quantified from the standard curves obtained in each run. The log copy number of the corresponding genes were converted to cell equivalents. For *B. thetaiotaomicron*, only one copy of the target gene is present in a cell, thus one copy number corresponds to one cell. Inhibition controls were prepared as detailed in ISO/TS 12869:2012. This involved using sample extracts and known quantities of *B. thetaiotaomicron* BT_2225 qPCR product to assess inhibition. All sample extracts were checked for qPCR inhibition based on the difference in corresponding threshold cycle values of the sample compared to a control containing molecular grade water. The r2 value was greater than 0.99 for all assays. The limit of quantification was less than 10 CFUs.
